# The Relationship Between Respiratory-Related Premotor Potentials and Small Perturbations in Ventilation

**DOI:** 10.3389/fphys.2018.00621

**Published:** 2018-05-30

**Authors:** Anna L. Hudson, Marie-Cécile Niérat, Mathieu Raux, Thomas Similowski

**Affiliations:** ^1^Neuroscience Research Australia and University of New South Wales, Sydney, NSW, Australia; ^2^Sorbonne Université, INSERM, UMRS1158 Neurophysiologie Respiratoire Expérimentale et Clinique, Paris, France; ^3^AP-HP, Groupe Hospitalier Pitié-Salpêtrière Charles Foix, Département d’Anesthésie Réanimation, Paris, France; ^4^AP-HP, Groupe Hospitalier Pitié-Salpêtrière Charles Foix, Service de Pneumologie et Réanimation Médicale, Paris, France

**Keywords:** readiness potential, Bereitschaftspotential, dyspnea, electroencephalography, EEG, respiration

## Abstract

Respiratory-related premotor potentials from averaged electroencephalography (EEG) over the motor areas indicate cortical activation in healthy participants to maintain ventilation in the face of moderate inspiratory or expiratory loads. These experimental conditions are associated with respiratory discomfort, i.e., dyspnea. Premotor potentials are also observed in resting breathing in patients with reduced automatic respiratory drive or respiratory muscle strength due to respiratory or neurological disease, presumably in an attempt to maintain ventilation. The aim of this study was to determine if small voluntary increases in ventilation or smaller load-capacity imbalances, that generate an awareness of breathing but aren’t necessarily dyspneic, give rise to respiratory premotor potentials in healthy participants. In 15 healthy subjects, EEG was recorded during voluntary large breaths (∼3× tidal volume, that were interspersed with smaller non-voluntary breaths in the same trial; in 10 subjects) and breathing with a ‘low’ inspiratory threshold load (∼7 cmH_2_O; in 8 subjects). Averaged EEG signals at Cz and FCz were assessed for premotor potentials prior to inspiration. Premotor potential incidence in large breaths was 40%, similar to that in the smaller non-voluntary breaths in the same trial (20%; *p* > 0.05) and to that in a separate trial of resting breathing (0%; *p* > 0.05). The incidence of premotor potentials was 25% in the low load condition, similar to that in resting breathing (0%; *p* > 0.05). In contrast, voluntary sniffs were always associated with a higher incidence of premotor potentials (100%; *p* < 0.05). We have demonstrated that in contrast to respiratory and neurological disease, there is no significant cortical contribution to increase tidal volume or to maintain the load-capacity balance with a small inspiratory threshold load in healthy participants as detected using event-related potential methodology. A lack of cortical contribution during loading was associated with low ratings of respiratory discomfort and minimal changes in ventilation. These findings advance our understanding of the neural control of breathing in health and disease and how respiratory-related EEG may be used for medical technologies such as brain-computer interfaces.

## Introduction

The respiratory muscles are unique because respiratory motoneurones are rhythmically depolarized by neural drive that originates from the permanently active automatic pacemaker centers in the ponto-medullary region. This drive can be supplemented or temporarily bypassed by inputs, for example, from the motor cortex during voluntary movements (for review see Butler et al., 2014). The neural control of self-paced movements is commonly assessed using electroencephalography recordings (EEG) as the presence of a *Bereitschafts* (readiness) or premotor potential in averaged EEG trials indicates activity in the supplementary motor area, premotor- and primary motor- cortices related to preparation and execution of voluntary movements (Shibasaki and Hallett, 2006). For respiratory motor control, Macefield and Gandevia (1991) were the first to demonstrate that a premotor potential precedes inspiration or expiration in voluntary self-paced brisk sniffs or exhalations, respectively. Importantly, they also demonstrated that a premotor potential over the cerebral cortex is not detected during resting breathing in healthy participants (Macefield and Gandevia, 1991), consistent with neural drive from the ponto-medullary region and not the cerebral cortex in this circumstance. However, premotor potentials are present during resting breathing in patients with congenital central hypoventilation syndrome (CCHS) (Tremoureux et al., 2014a) or amyotrophic lateral sclerosis (ALS) (Georges et al., 2016). This suggests that additional inputs to the respiratory muscles, i.e., a ‘cortical contribution,’ are required to maintain, or an attempt to maintain, resting ventilation in these patients. Insufficient neural drive to the respiratory muscles from the automatic respiratory centers during wakefulness has important consequences on hypoventilation during sleep (e.g., Arnulf et al., 2000). During wakefulness, the cortical compensation of insufficient automatic breathing drive has been associated with dyspnea (e.g., Morawiec et al., 2015; Georges et al., 2016).

To date in healthy participants, the premotor potential technique has demonstrated a cortical contribution to respiratory muscle control during ballistic sniff or exhalation movements (see above) or during loaded breathing with moderate (∼10 or more cmH_2_O) inspiratory or expiratory threshold or resistive loads (Raux et al., 2007b; Tremoureux et al., 2010; Morawiec et al., 2015; Hudson et al., 2016). A cortical contribution can also be detected prior to inspiration during other ‘tasks’ or conditions such as speech (Tremoureux et al., 2014b) or the persistence of ventilation in spite of hyperventilation-induced hypocapnia (Dubois et al., 2016). The ballistic maneuvers and moderate loads are beneficial experimentally as ‘positive controls’ in order to demonstrate that premotor potentials are typically absent during resting breathing in healthy participants and to simulate an imbalance between the load on- and capacity of- the respiratory muscles as seen in respiratory and neurological disease (Tremoureux et al., 2014a; Hudson et al., 2016; Navarro-Sune et al., 2016). However, it is not known if small voluntary increases in ventilation or smaller load-capacity imbalances (that generate an awareness of breathing, but aren’t necessarily dyspneic) are sufficient to give rise to respiratory premotor potentials in healthy participants. This is important to understand the mechanisms that cause a cortical contribution to maintain resting breathing in patients with a respiratory load-capacity imbalance.

Given that ventilation during speech and hyperventilation-induced hypocapnia are associated with premotor potentials in healthy participants (see above), we hypothesized that premotor potentials would be present prior to small voluntary and load-induced perturbations to ventilation. Thus, we recorded EEG during large voluntary breaths and when breathing through a ‘low’ inspiratory threshold load (ITL). To determine if breath-to-breath changes in cortical input to breathe can be detected using EEG, we compared large voluntary breaths and smaller non-voluntary breaths, interspersed in the same trial. Functional magnetic resonance imaging shows activation across a large number of brain regions following single breath inspiratory loads which suggests there are breath-to-breath changes in activation of ‘voluntary respiratory networks’ (Raux et al., 2013), but it is not known if these can be detected by EEG in the form of a premotor potential. Detection of breath-to-breath changes in the neural control of breathing in healthy participants using EEG will inform the continued development of a brain-ventilator interface that proposes the use of EEG to detect transient respiratory-related changes in cortical activation in critically ill patients (Navarro-Sune et al., 2016).

## Materials and Methods

The studies were carried out in 15 participants (7 female) aged 19–25 years. The subjects gave informed written consent to the procedures which conformed with the Declaration of Helsinki and were approved by the *Comité de Protection des Personnes Ile-de-France VI*, Groupe Hospitalier Pitie-Salpetriere, Paris, France. Electroencephalographic activity (EEG) was recorded during 2 different primary respiratory conditions: ‘big breaths’ (∼3× tidal volume) or ‘low loads’ (an ITL set to ∼7 cmH_2_O). A cortical contribution to breathing in these conditions was assessed by the presence of an inspiratory Bereitschaft (readiness) or premotor potential. Positive and negative control conditions, i.e., conditions known to be associated with and without an inspiratory premotor potential in healthy participants, were sniffs and resting unloaded breathing, respectively. For 12 participants, premotor potential data during sniffs and resting breathing only have been reported previously (Hudson et al., 2016).

### Experimental Set-Up and Protocol

Electroencephalography and respiratory variables were measured as previously described (Hudson et al., 2016). Briefly, participants breathed through a mouthpiece connected to a pneumotachograph (3700 series; Hans Rudolph) and two-way valve (2700 series; Hans Rudolph) and airflow and mouth pressure were measured. EEG was measured from central scalp locations (Cz and FCz), the earlobes and under the right eye to monitor electrooculographic activity using an active electrode system (ActiCap; Brain Products). EEG signals were time locked to respiratory signals with simultaneous digital trigger pulses. Surface electromyographic (EMG) recordings were made from the scalene muscles on the right side with adhesive electrodes to assess if premotor potentials were associated with increases in inspiratory muscle activity as previously observed (Georges et al., 2016). One electrode was placed on the posterior triangle of the neck, posterior to the border of the sternocleidomastoid muscle at the level of the cricoid cartilage, and the second electrode was placed ∼2 cm caudally over the muscle. Respiratory signals and EMG were sampled at 2 kHz and EEG at 2.5 kHz (PowerLab, AD Instruments).

**FIGURE 1:**
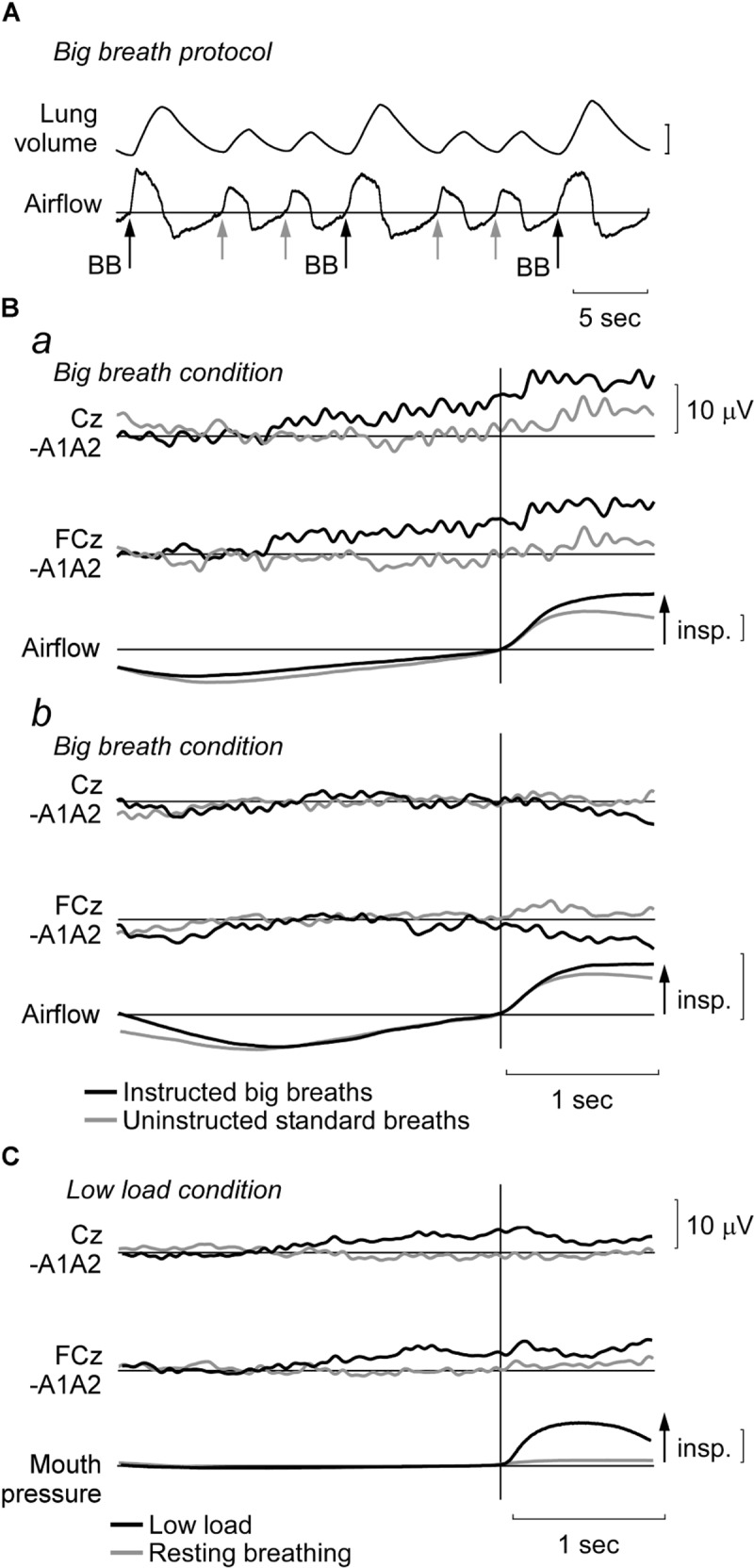
Representative recordings and EEG data during big breath and low load conditions. **(A)** In a trial of unloaded breathing, participants were randomly instructed to perform a ‘big breath,’ i.e., aim to double tidal volume. For analysis, waveform averages were made from EEG segments from these instructed big breaths (BB; black arrows) and the uninstructed standard breaths in these trials (gray arrows). Vertical lung volume calibration: 1 l. **(B)** Average EEG traces at Cz and FCz and airflow for two participants during the big breath condition. Typical signals are shown for a participant (*Ba*) with a premotor potential during the instructed big breaths (black traces), but no potential during the uninstructed standard breaths (gray traces). For a different participant (*Bb*), a premotor potential was not observed in either condition. Vertical airflow calibration: 0.5 l/s. **(C)** Average EEG traces (as above) and mouth pressure during low inspiratory threshold load condition (black traces) and resting breathing (gray traces) for a participant in whom a premotor potential was detected in the low load condition. Vertical mouth pressure calibration: 10 cmH_2_O.

Ten of the fifteen participants performed the big breath condition. Here, during a trial of resting unloaded breathing, participants were instructed to take larger breaths of approximately twice the tidal volume of resting breaths (**Figure 1**). They were instructed by an experimenter during the preceding expiration and did not have visual feedback of lung volume. Instructions to take big breaths were given randomly, every 2–3 breaths. Eight of the fifteen participants performed a low load condition during which an ITL set to ∼7 cmH_2_O was attached to the inspiratory port of the two-way valve in the respiratory apparatus. At the end of this condition, subjects (*n* = 6) rated their level of respiratory discomfort during the condition using a visual-analog scale that ranged from zero: “no breathlessness or discomfort” to ten: “maximal breathlessness or discomfort.” The other respiratory conditions, i.e., the positive and negative controls of self-paced voluntary sniffs and resting breathing, are described in Hudson et al. (2016) and were performed by all participants in the same recording session as the big breath or low load conditions. For 12 participants, premotor potential data during sniffs and resting breathing were reported previously (Hudson et al., 2016), but 3 participants had not performed the sniff and resting breathing conditions.

### Data Analysis

Offline, EEG recordings were resampled at 250 Hz, referenced to linked-ear electrodes and band-pass filtered (0.05–10 Hz). EEG was divided into 3.5-s segments, with 2.5 s prior to and 1 s after the onset of inspiration, based on airflow (big breaths) or negative mouth pressure (ITL). In the big breath condition, breaths were identified as either ‘instructed big breaths’ (i.e., the breaths when participants were told to increase tidal volume) or ‘uninstructed standard breaths’ (i.e., breaths in the trial that were of usual tidal volume as participants were not given directive). EEG segments were visually inspected and rejected from the average if they exhibited artifact, e.g., large deviations from baseline or intense EOG activity and the remaining segments were averaged. For the big breaths condition, 59 ± 9 (mean ± SD, range 50–78) and 76 ± 22 (range 55–123) segments were averaged for the instructed big breaths and uninstructed standard breaths, respectively. This corresponded to 87% (range 70–97%) and 82% (range 60–99%) of all segments recorded for the instructed big breaths and uninstructed standard breaths, respectively. For the low load condition, 90 ± 13 segments (range 80–109), or 75% (range 63–87%) of all segments, were averaged. For the three participants who had not performed the sniff and resting breathing conditions previously, 89 ± 4 (range 86–93) and 119 ± 46 (range 68–156) segments were averaged, respectively. This was 93% (range 89–97%) and 77% (range 56–97%) of all segments recorded for sniffs and resting breathing, respectively.

Average EEG traces at Cz and FCz derivations were then de-identified and examined for the presence of an inspiratory premotor potential, i.e., a slow increasingly negative shift in the Cz or FCz EEG signal starting from ∼2–0.5 s before inspiration. The signals were judged by three assessors (authors AH, MR, and TS), all blinded to the condition and participant during assessment, with incidence determined by majority. If present, the latency of each premotor potential was measured as the duration (in ms) between the onset of EEG negativity and the start of inspiration and the amplitude (in μV) of the EEG negativity at the start of inspiration was measured. Respiratory variables were measured for all breaths using a semi-automated script and averaged for each condition. Predicted maximal inspiratory pressure was calculated (Harik-Khan et al., 1998). To measure scalene EMG, the root mean square (rms, time constant 50 ms) of the EMG signal was computed and ensemble averaged for all breaths in each condition (see Hug et al., 2006). The area and amplitude of phasic inspiratory EMG was measured between the onset of inspiratory activity (i.e., the increase in activity from any tonic activity during expiration) and the end of inspiration. To compare between subjects, the inspiratory EMG area and amplitude in instructed big breaths, uninstructed standard breaths and low load were normalized to these measures for the same subject during the trial of resting breathing. Respiratory variables and EMG were also determined for signals recorded during resting breathing for all subjects as these measures were not reported with the previously published EEG data (Hudson et al., 2016).

### Statistics

Group data are presented as mean (SD) or median [IQR] for parametric and non-parametric data, respectively. The H-statistic (degrees of freedom; df) is shown for unpaired data and the F-statistic or *Z* value (df) is shown for repeated measures and paired data, respectively.

To compare respiratory variables between instructed big breaths and uninstructed standard breaths in the big breath condition and with resting breathing, a one-way repeated measures analysis of variance (ANOVA) was used, with pairwise *post hoc* testing using the Holm-Sidak test. Normalized EMG was compared between instructed big breaths and uninstructed standard breaths with a paired *t*-test. A Kruskal–Wallis one-way analysis of variance (ANOVA) on Ranks with pairwise *post hoc* testing using the Tukey’s test was performed to compare the incidence of inspiratory premotor potentials between conditions. Respiratory variables and scalene EMG in recordings with and without a premotor potential were compared using *t*-tests.

## Results

**FIGURE 2:**
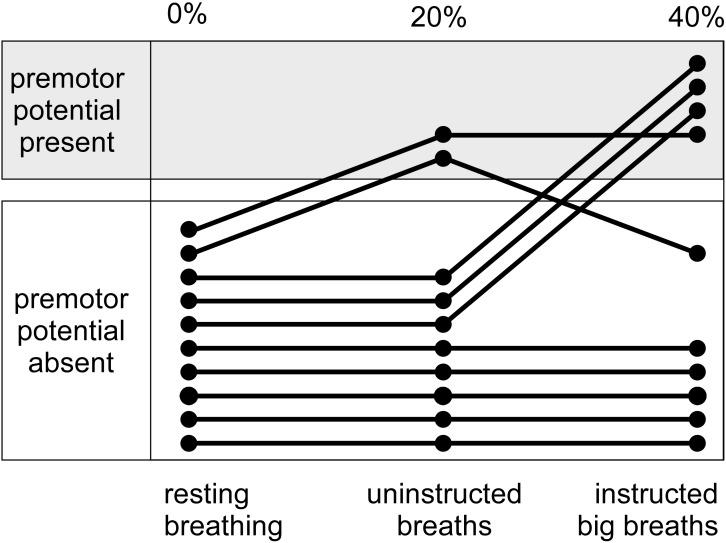
Incidence of premotor potentials for interspersed instructed big breaths and uninstructed standard breaths during a trial of unloaded breathing. Shows, for individual participants, the distribution of premotor potentials during instructed big breaths and uninstructed standard breaths, compared to a trial of resting breathing.

For the big breath condition, ‘instructed big breaths’ and ‘uninstructed standard breaths’ were interspersed as participants were instructed randomly to take larger breaths, and elsewise were uninstructed and could breathe as usual. Average EEG was compared for instructed big breaths and uninstructed standard breaths, during which respiratory variables and inspiratory scalene EMG were different (see **Table 1**). **Figure 1** shows typical examples of averaged EEG signals for instructed big breaths and uninstructed standard breaths performed in the same trial for two participants. Premotor potentials were present in four of ten participants during instructed big breaths and in two participants during uninstructed standard breaths (one participant in common; see **Figure 2**). For the group, the incidence of premotor potentials varied between conditions [*H*_(3)_ = 22.8, *p* < 0.001]. There was no difference in the incidence between big breaths (40%) and resting breathing (0%) or sniffs (100%), but as reported previously (Hudson et al., 2016), the incidence varied between sniffs and resting breathing (*p* < 0.05). The incidence was also lower in uninstructed standard breaths (20%), compared to sniffs (*p* < 0.05). The amplitudes and latencies of premotor potentials in instructed big breaths and uninstructed standard breaths are summarized in **Table 2**.

**Table 1:** Mean (SD) or median [IQR] respiratory variables and inspiratory EMG during EEG recordings of instructed big breaths, uninstructed standard breaths, and resting breathing.

	Instructed big breaths	Uninstructed standard breaths	Resting breathing	Statistic and *P*-value
Tidal volume (l)	1.39 (0.48)^∗†^	0.48 (0.15)	0.49 (0.07)	*F*_(2,18)_ = 47.79, *p* < 0.001
Insp. time (s)	4.05 (1.04)^∗†^	2.11 (0.58)	1.78 (0.35)	*F*_(2,18)_ = 58.00, *p* < 0.001
Mean flow (l/s)	0.38 (0.18)^∗^	0.24 (0.08)	0.29 (0.04)	*F*_(2,18)_ = 5.95, *p* = 0.01
Mouth pressure (cmH_2_O)	1.95 (0.86)^∗†^	1.33 (0.34)	1.49 (0.30)	*F*_(2,18)_ = 6.00, *p* = 0.01
Scalene EMG area (norm.)	7.08 [5.57–26.15]	1.64 [1.04–2.88]	–	*Z* = -2.8, *p* < 0.01
Scalene EMG amplitude (norm.)	5.10 [2.48–14.87]	1.52 [1.18–2.16]	–	Z = -2.8, *p* < 0.01

*Statistic and p-values are shown. EMG values are normalized (norm.) to those in resting breathing. ^∗^significant difference to uninstructed standard breaths*, **^†^***significant difference to resting breathing.*

**Table 2:** Median [IQR] amplitude and latency of premotor potentials during EEG recordings.

	Instructed big breaths	Uninstructed standard breaths	Low load
Amplitude FCz (μV)	1.86 [1.64–2.38]	2.88 [2.28–3.49]	4.88 [4.56–5.20]
Amplitude Cz (μV)	3.24 [2.17–4.23]	3.02 [2.97–3.06]	5.43 [5.34–5.52]
Latency FCz (ms)	1325 [1300–1400]	1700 [1550–1850]	1575 [1488–1663]
Latency Cz (ms)	1325 [1300–1388]	1700 [1550–1850]	1600 [1500–1700]

*For FCz and Cz, the parameters of premotor potentials for instructed big breaths (*n* = 4 participants), uninstructed standard breaths (*n* = 2), and low loading (*n* = 2) are shown*.

Breathing through a low load was associated with a premotor potential in two of the eight participants. For the group, the incidence of premotor potentials varied with condition [*H*_(2)_ = 17.1, *p* < 0.05]. The incidence was 25% in low load condition, similar to that in resting breathing (0%; *p* > 0.05), but significantly less than in the sniff condition (100%; *p* < 0.05). The amplitudes and latencies of premotor potentials are shown in **Table 2**. For the breaths during the ITL condition, mouth pressure averaged 7.50 (2.85) cmH_2_O, tidal volume was 0.64 (0.21) l, inspiratory time was 2.39 (0.78) s, mean flow was 0.31 (0.18) l/s and ventilation was 7.08 (2.04) l/min. Normalized to resting breathing for each subject, inspiratory scalene EMG area and amplitude during ITL averaged 3.49 (1.78) and 2.90 (1.88), respectively. For the six subjects who rated the respiratory discomfort associated with the low load condition, it averaged 1.1 (1.2) on a scale from zero-ten and was not different between those with (1.7 [0.0–3.3]) and without (1 [0.3–1.2]) a premotor potential (*p* = 0.80).

**FIGURE 3:**
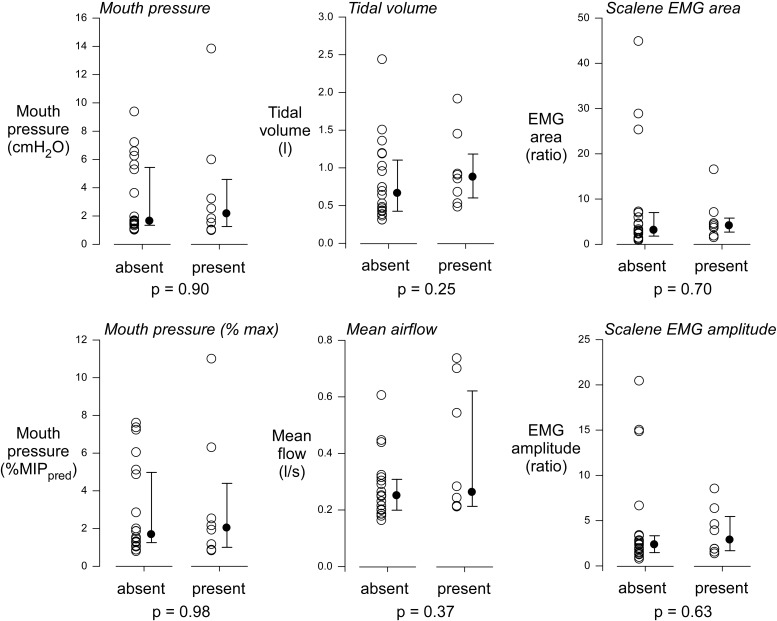
Comparison of respiratory and EMG signals with and without premotor potentials. For EEG averages from instructed large breaths, uninstructed standard breaths, and low load conditions, a premotor potential was detected in 8 instances. The respiratory variables and EMG measures during these recordings were similar to those in the 20 instances when EEG averages did not evidence a premotor potential. MIP_pred_, predicted maximal inspiratory pressure.

For big breath and low load conditions together, a premotor potential was detected in eight instances (four in instructed big breaths, two in uninstructed standard breaths, and two in the low load condition) in seven participants. As shown in **Figure 3**, the respiratory variables and scalene EMG in the trials with a premotor potential were similar to those without a premotor potential.

## Discussion

This is the first study to investigate if small perturbations to ventilation are associated with respiratory-related premotor potentials in healthy participants. The incidence of potentials in large breaths (∼3× tidal volume) and low loads (∼7 cmH_2_O) was similar to that in resting breathing. This suggests that, in contrast to other tasks such as speech or the persistence of ventilation in spite of hyperventilation-induced hypocapnia, the voluntary command to transiently (i.e., single breath) increase breath size or to maintain small increases in inspiratory pressure with an inspiratory threshold load are not associated with respiratory-related cortical contribution in healthy participants, as detected using event-related potential methodology.

### Methodological Considerations

The event-related potential technique used here is sensitive enough to detect premotor potentials in these types of experimental conditions as evidenced by their detection in resting breathing in patient populations (Tremoureux et al., 2014a; Launois et al., 2015; Georges et al., 2016) and in ‘pre-phonatory’ breaths in speech with similar tidal volume to that in the larger breaths here (Tremoureux et al., 2014b). The number of segments available for the large breath analysis was less than 80, as is typically used to detect respiratory-related premotor potential from average EEG signals (e.g., Raux et al., 2007b). Combined with the presumably smaller signal to noise ratio in this task, i.e., compared to sniffs (Tremoureux et al., 2014b), we may have underestimated the incidence of premotor potentials in the instructed larger breaths and uninstructed standard breaths. However, for the low load condition, 80 or more artifact-free segments were available for each participant and the incidence in this condition was also similar to that in resting breathing. Our observations do not completely rule out some degree of cortical activation, but indicate that if present, it is too weak to be readily and consistently detected by an event-related approach. Alternative methodology to detect cortical activation such as time-frequency maps are not likely to offer better sensitivity given we previously demonstrated that premotor incidence was superior to time-frequency maps to discriminate between resting breathing and moderate inspiratory threshold loading (Hudson et al., 2016).

For the large breath condition, participants were instructed during expiration to increase tidal volume on the subsequent breath, but following this general instruction, participants could choose when and how they made the big breath. Therefore, our protocol in the large breaths condition was not true “volitionality” (Deecke and Soekadar, 2016), but as argued by Schultze-Kraft et al. (2016) who used a similar protocol to investigate the temporal relationship between the readiness potential and the cancelation of movements, nor was it a contingent negative variation (CNV) paradigm (Haynes and Schultze-Kraft, 2016). Thus, in the present study, it was reasonable to expect that a readiness potential would be present prior to the instructed large breaths and to use event related potential methodology to detect it.

Evaluation of the relationship between the presence of a premotor potential and respiratory discomfort is limited in the present study as participants who performed the big breath condition were not asked to rate respiratory discomfort. The current protocol, in which instructed big breaths were interspersed with uninstructed standard breaths, prohibited this assessment. A protocol of sustained voluntary hyperventilation could be used to investigate the relationship between premotor potentials and discomfort with voluntary increases in breath size, and indeed to determine if continuous (i.e., not transient) increases in tidal volume elicit premotor potentials in healthy participants. Compared to resting breathing, sustained voluntary increases in breathing frequency are associated with greater respiratory-EEG coherence from numerous brain areas, including the frontal, precentral, and insular cortices, as measured with intracranial EEG in the non-affected brain areas of epileptic patients (Herrero et al., 2017).

### Premotor Potentials, Ventilation and Respiratory Discomfort

Our data support the emerging theory that in the absence of an automatic breathing defect (see below), for a cortical activation sufficient to give rise to premotor potentials breathing must probably be dyspneic rather than be just a conscious sensation or awareness of breathing. Respiratory sensation arises from feedback from multiple afferents sources in the periphery (i.e., reafference, from for example, the lungs and respiratory muscles), but also in the form of corollary discharge, i.e., an ascending copy of the neural drive to breathe that originates from the medulla and/or motor cortex (Parshall et al., 2012). Typically, with increases in ventilation, there is more corollary discharge, of either medullary or cortical origin, or both, as well as increased afferent feedback, e.g., from the lungs with greater expansion in inspiration. However, the magnitude of corollary discharge with increased drive to breathe is not necessarily matched to an increase in afferent feedback from the periphery, e.g., if there is impaired respiratory mechanics or muscle weakness in respiratory or neurological disease. Dyspnea, the sensation of breathlessness, can be classified as sensations of (i) work or effort, (ii) chest tightness, or (iii) air hunger/unsatisfied inspiration. It is thought that dyspnea occurs not only from an awareness that breathing has increased, but rather from the “perception that the drive to breathe is not being matched by adequate pulmonary ventilation” (Parshall et al., 2012), i.e., a ‘mismatch’ between corollary drive and afferent feedback.

In healthy participants, respiratory-related premotor potentials are typically absent (incidence of 0–10%) during resting breathing, but present (incidence of 50–100%) during threshold or resistive loads of 10 cmH_2_O or more, as well as in large ballistic movements like voluntary sniffs (Macefield and Gandevia, 1991; Raux et al., 2007b; Morawiec et al., 2015; Hudson et al., 2016). Although the loading and ballistic conditions are typically associated with increases in ventilatory variables such as tidal volume and/or mouth pressure, respiratory-related premotor potentials in healthy participants are not interrelated with increases in ventilation. In addition to our findings, further evidence that increases in ventilation alone do not evoke premotor potentials in healthy participants, is the nil-to-negligible incidence of cortical activation during hypercapnic breathing and exercise, i.e., comparable to the incidence in resting breathing (Raux et al., 2007b; Jutand et al., 2012; Tremoureux et al., 2014a). Hypercapnic gas mixtures are likely to increase ventilation from stimulation of the automatic respiratory centers and thus a lack of cortical contribution would be expected. Of note, the dyspnea in the hypercapnic condition is described as ‘air hunger,’ rather than ‘effort’ as in inspiratory threshold loading (Raux et al., 2007b). Consistent with no direct relationship between premotor potentials and increases in ventilation in healthy participants, we have shown that voluntary large breaths and low loads also do not strongly elicit of respiratory-related premotor potentials. Furthermore, for these conditions, the respiratory variables were similar in trials with and without a premotor potential.

Along these lines, there can be an increase in the incidence of respiratory-related premotor potentials in healthy participants that is not associated with changes in ventilation. For example, during non-invasive pressure support ventilation in healthy participants adjusted incorrectly to “discomfort” to model “ventilator fighting,” there is 100% incidence of premotor potentials (Raux et al., 2007a). The respiratory variables during these recordings were similar to those when the support ventilation was correctly adjusted (i.e., “comfort”) when the incidence of premotor potentials was only ∼29%. Rather than altered ventilation, the different conditions of “discomfort” and “comfort” were associated with changes in ratings of the intensity of respiratory discomfort, of the work/effort type, and scalene muscle EMG (Raux et al., 2007a). Consistent with this, the incidence of premotor potentials in the low load condition was similar to resting breathing in the present study and the ratings of dyspnea were low. However, no difference in inspiratory scalene EMG in the trials with and without premotor potentials was observed in the larger breaths and low load conditions.

Data from patient groups support the concept that respiratory-related premotor potentials may be related to “discomfort,” rather than increased afferent feedback and an awareness of breathing because ventilation is higher. Approximately 60% of patients with ALS exhibit a premotor potential in resting breathing and respiratory discomfort is rated higher in these patients, compared to those who do not have a premotor potential (Georges et al., 2016). Furthermore, in the sub-group with a premotor potential, there are parallel reductions in respiratory discomfort and premotor potential incidence during non-invasive ventilation (Georges et al., 2016). Although from a separate cohort of ALS patients, non-invasive ventilation typically increases tidal volume (Magalhaes et al., 2016), which suggests the reduction in premotor potential observed by Georges et al. (2016) was not associated with decreases in ventilation (and the associated reduction in respiratory afferent feedback). Impaired automatic drive to breathe in CCHS is associated with a 100% incidence of premotor potentials to maintain ventilation during resting breathing in wakefulness (Tremoureux et al., 2014a). Although the corresponding ventilatory data during these EEG recordings was not reported, the tidal volume and ventilation during resting breathing in wakefulness has been reported to be similar for CCHS and healthy participants (Ranohavimparany, 2012). CCHS patients do not experience dyspnea during resting breathing in wakefulness (Similowski, unpublished observations) or even during exposure to high levels of CO_2_ (Shea et al., 1993). However, as suggested by a case study in a CCHS patient, there may be other ‘negative’ effects of continuous cortical control of breathing, such as impaired performance in cognitive tasks (Sharman et al., 2014). Here, larger voluntary breaths that were almost three times the tidal volume of resting breathing resulted in a premotor potential in only 40% of healthy participants. Given the high incidence of premotor potentials in ALS and CCHS which were likely to be associated with breaths of usual or lower tidal volumes, our findings support the concept that the presence of a premotor potential in breathing may be due to a mismatch between central neural drive and afferent feedback from the respiratory system, rather than just due to an awareness of breathing because of increased afferent feedback. This mismatch may manifest as abnormal respiratory perception, i.e., dyspnea, or cognitive impairment, but it remains to be determined whether premotor potentials are a neurophysiological manifestation of the mismatch between one or both (i.e., medullary and cortical) forms of corollary discharge and reafference.

### The Clinical Relevance and Application of Respiratory Premotor Potential Methodology

Of course the load-capacity imbalance evoked in healthy participants using external inspiratory threshold loads is not permanent like those in disease (although it may fluctuate in disease, e.g., with exacerbations in chronic obstructive pulmonary disease and asthma). However, premotor potentials in healthy participants do persist during ‘chronic’ or sustained exposure (1 h) of an inspiratory threshold load of 17 cmH_2_O or more (Tremoureux et al., 2010). Using functional magnetic resonance imaging, although the network of brain areas associated with continuous inspiratory threshold loading was shown to be limited compared to single-breath load exposure, 3 min of continuously loaded breaths (∼10 cmH_2_O inspiratory threshold load) was associated with persistent activation in numerous regions, including the supplementary area (Raux et al., 2013) (see also Gozal et al., 1995). Here, with ∼10 min of a ∼7 cmH_2_O load, there may have been greater ‘automatization’ and thus no physiologically significant cortical activation (i.e., as a premotor potential) to detect in the majority of our participants. That there was a limited degree of cortical contribution to the breaths with a low load is corroborated by the fact that ventilation during this condition here (∼7 L/min) is similar to that in resting breathing (Hudson et al., 2016). Given that healthy participants typically over-ventilate in response to an external load in wakefulness, but that this response is abolished and even reversed, during sleep (Henke et al., 1992), the current data of a lower incidence in cortical activation and no change in ventilation are consistent with minimal cortical activation. Immediate changes to the breathing pattern and diaphragm EMG with unexpected presentation of loads near or below the perceptual threshold suggests the response to loading, at least for these very low loads, are not due to perceptual response, i.e., are independent of cortical networks (Daubenspeck and Bennett, 1983; Kobylarz and Daubenspeck, 1992). Even for loads above perceptual threshold, but still of low magnitude (i.e., similar to that used here), the ventilatory response is moderate and transient (Freedman and Weinstein, 1965; Yan and Bates, 1999) compared to ‘bigger loads,’ i.e., those associated with reliable evidence of cortical activation in the form of premotor potentials (Raux et al., 2007b). Given the cortical response to low loads is likely to be transient, or at least diminished in its magnitude (see Raux et al., 2013), a method with superior time resolution (see below) is required to detect any cortical changes associated smaller loads, especially given these are more relevant for pathophysiological conditions.

Compared to the low load, the larger breaths condition was different in that ‘instructed large breaths’ were interspersed with ‘uninstructed standard breaths.’ Therefore, there was breath-to-breath variation which is likely to be associated with less automatization and a different preparatory state to loading. Preparatory state has been identified as a factor that influences the premotor potential (Shibasaki and Hallett, 2006). Here, the incidence of premotor potentials was 40% for large breaths and 20% non-instructed standard breaths with the interspersed protocol compared to 25% in the low load. However, direct comparisons of the incidence between the large breaths and low load conditions is not appropriate due to differences in the ventilation changes, i.e., disparities in tidal volume changes with mouth pressure with and without an inspiratory threshold load. To confirm that breath-to-breath fluctuations in respiratory control evoke more cortical activation compared to continuous increases in cortical activation, future studies should compare cortical EEG between continuous- and interspersed- augmented breaths and between continuous- and single breath-loading (Raux et al., 2013). The ability to detect transient changes in respiratory-related cortical activation using EEG is critical for the continued development of the brain-ventilator interface (Navarro-Sune et al., 2016) that aims to use EEG to optimize mechanical ventilation in critically ill patients. However, given the load-capacity imbalance in critically ill patients can change quickly due to changes in respiratory mechanics or neural activity (e.g., due to changes in sedation or pain), the use of event-related potentials to detect changes in respiratory control is limited by poor time resolution and continuous connectivity approaches are preferable (Hudson et al., 2016; Navarro-Sune et al., 2016).

## Conclusion

We have demonstrated that in contrast to respiratory and neurological disease, there is no significant cortical contribution to increase tidal volume or to maintain the load-capacity balance with a small inspiratory threshold load in healthy participants as detected using event-related potential methodology. The lack of cortical contribution during loading was associated with low ratings of respiratory discomfort and minimal changes in ventilation. This infers that the changes in the neural control of breathing, i.e., cortical activation, in patients maintain ventilation during resting breathing in wakefulness, but the consequences of this physiological adaptation are likely to include dyspnea and cognitive impairment during wakefulness and hypoventilation during sleep.

## Author Contributions

AH, MR, and TS contributed to study conception and design. AH and M-CN collected the data. AH analyzed the data and drafted the manuscript. All authors interpreted the analyzed data and critically revised the manuscript. All authors approved the final version of the manuscript and all persons who qualify for authorship are listed.

## Conflict of Interest Statement

The authors declare that the research was conducted in the absence of any commercial or financial relationships that could be construed as a potential conflict of interest.
